# REACHING THE UNREACHED IN SUNDERBANS

**Published:** 2017-02-10

**Authors:** Asim Sil

**Affiliations:** 1 Medical Director, Vivekananda Mission Ashram Netra Niramay Niketan.

## Background

The Sunderbans is situated in the Ganges delta, bordering the Bay of Bengal, with a large component being in Bangladesh. The Indian part, which is in West Bengal State has 106 islands and 24 (Parganas) districts. People live on 52 islands and the adjacent mainland, with the uninhabited areas being mainly mangrove forests.

The Sunderbans is a very challenging areas to live in, and the area is prone to natural disasters such as typhoons and flooding. The population of 19 blocks of Sunderban was estimated at 4.7 million in 2011. It is an area of extreme poverty and ill health exacerbated by access difficulties. Almost half of the population (47%) are historically marginalized groups such as Scheduled Castes and Tribes. More than 40% of households live below the poverty line and 13% are officially declared as the “poorest of the poor”.

The main occupations are farming and fishing. Cultivation depends on rain water as the river water has high salinity, and over half of those engaged in farming are landless laborers. To protect fields from salty river water high embankments are built around cultivated land.

Out migration of those of working age to cities and towns is very high and the worst social problem is human trafficking. Areas which have good infrastructure which connect communities to the mainland have higher socioeconomic status than island communities where transport relies on the waterways.

As survival is the main issue, education and health are not given high priority. For example, despite high primary school enrolment, there is very high non-attendance in upper primary levels.^1^ Availability of health care facilities varies from less than one to five per 100,000 population,^3^ and the morbidity rate is higher in Sunderban than the state average. Children are three times more prone to respiratory diseases and communicable diseases are highly prevalent. People who collect honey in the forests or catch fish are under constant threat of attacks by animals and snake bites.^2^

NGO hospitals are the major service providers but may also Sunderban indicate poor utilization of public facilities. Major part of the Indian Sunderbans belongs to South 24 Parganas District where 83% and 14% cataract surgeries are done by NGO and Government Hospitals respectively.

## Sunderban's Eye Health Service Strengthening Project

Standard Chartered Bank, under the “Seeing is Believing” initiative is supporting Sightsavers to implement the “Sunderbans Eye Health Service Strengthening Project”. The objective of the five year project, 2013-2018, is to contribute to the elimination of avoidable blindness in the area.

## Baseline Study on Eye Health in Sunderban

In order to assess eye health status and health seeking behavior, a population based survey among individuals aged 40 years and above was conducted as the initial step. The survey identified 3,388 eligible individuals living in 19 blocks 2,854 (84.2%) of whom were examined. There was higher non response amongst males due to occupational migration. The prevalence of blindness using the World Health Organization definition (presenting VA<3/60 in the better eye) was 1.9% (2.1% among those aged 50 years and above). Using the Indian (NPCB) definition (presenting VA<6/60 in the better eye) the prevalence was 6.7% (10.0% amongst 50+). The prevalence of blindness was higher among females (8.0%) than males (5.6%). The prevalence of severe visual impairment (presenting VA<6/60 – 3/60) was 4.8% (7.2% among the 50+).

The prevalence of blindness in Sunderbans was 1.88% (NPCB definition) which is almost 40% higher than the national average (1.36%).^4^ Amongst those aged 40+, 83.8% of blindness was due to cataract, 12.0% due to refractive errors and 4.2% due to other causes. The commonest cause of blindness among the 50+ population was cataract (83.4%) being higher than the 77.5% reported from a RAAB survey (2007) in West Bengal.^4^ Cataract surgical coverage was less than 50%, i.e. a large proportion of cataract-blind are still unreached. Women had a higher prevalence of blindness, higher proportion of cataract blindness and lowercataract surgical coverage than men.

Untreated cataract is the major cause of visual impairment at all levels (VA<3/60, VA<6/60 and VA<6/18 – best corrected VA or pinhole) of visual acuity. Overall, 1.2% of the total population is bilaterally blind due to cataract, and another 0.9% are blind in one eye. Women are disproportionately affected by cataract blindness both bilaterally (1.5% vs 1.0%) and unilaterally (1.0% vs 0.8%).

In total, nearly 11% of eyes in the sample were affected by cataract at VA<6/18 or less. This was greater among women (12.4%) than men (9.4%). Among people aged over 50, this proportion of cataract eyes increased to 18.5%.

The commonest reason given for not undergoing cataract surgery was ‘no felt need’ (30.8%), with underlying reasons being ‘old age’, ‘normal vision in other eye’ and ‘other competing priorities’. Amongst men, ‘cost of surgery’ was the next most common reason while women reported ‘lack of awareness about services.’

75.2% of the sample had presbyopia but less than half (46.2%) had access to near correction. More than half (54%) were not even aware that they could benefit from spectacles. Financial reasons were the most commonly reported barrier for not getting a check-up for glasses (51.4%). Broken or lost glasses were the most common reason (38.9%) for discontinuation of spectacle use. People are willing to pay INR 30 for check up and INR 100 for the glasses.^5^,^6^

## Baseline Study on Eye Health in Sunderban

Sightsavers is partnering with three eye care institutions (Southern Health Improvement Samity; Sunderban Social Development Centre and Vivekananda Mission Ashram, Chandi Branch) located near Sunderbans who are already providing services in the region. The Government Health Department is another partner. Both the facilities of Vivekananda Mission Ashram Netra Niramay Niketan are used as the training and referral centre.

## Human resource development

The core strategy of the initiative is to use local human resources to strengthen the eye care service because health professionals from outside are not likely to stay in such a difficult location. Local young people have been trained as Vision Technicians (VT) and Community Health Workers (CHW).

## Establishing Vision Centres

Seventeen Vision Centres have been established and are managed by trained VTs who perform refraction, recognize cataract and other conditions, referring cases to the NGO or Government hospitals. Spectacles are provided atan affordable or subsidized cost. Each centre has an optical dispensing unit which is supported by an optical laboratory at the base hospital. All these are stand-alone centres for eye care only. Two vision centres are being established within government PHCs.

## Awareness generation activities

Trained CHWs and VTs constantly engage in a range of awareness generation activities using IEC materials in group meetings and one-to-one counseling.

## Direct Service Delivery

The hospitals undertake outreach eye screening camps in interior locations in Sunderbans. The CHWs and VTs also conduct eye examination of children in schools near the vision centres where they provide free spectacles. People who need cataract surgery are taken to the base hospital and the follow up is arranged at the vision centre. This entire service is offered free of cost to patients.

## Strengthening the existing health system

In Sunderbans there are two Sub-Division Government hospitals with facilities for eye surgery. Efforts are underway to improve the volume and quality of cataract surgery through training. The government sub-divisional hospitals in Sunderbans are poorly managed, conducting less than 100 cataract surgeries annually. The project plans a facility survey, to enhance capacity, training on protocol and cataract management and thus hold hands to improve services locally.

Rural Medical Practitioners are important health providers in remote areas and there are plans to train 2,520 of these practitioners in primary eye care and proper referral.

Accredited Social Health Activists (ASHAs) and Auxiliary Nurse Midwives (ANMs) are workers at the grassroots level. 930 health workers of these cadres are being trained in identification of cataract and to create awareness.

Finding children with cataract continues to be challenging in Sunderban. A Higher proportion of boys with cataract was found and this could be due to two reasons. One is the health seeking behavior of the community and traumatic cataracts are greater more among boys.

## Challenges and way forward

Gaining the trust of the community was an initial challenge as some had had unpleasant experiences from other eye care providers. The quality and the price of spectacles, and poor quality of clinical services and cataract surgery were the main issues.

Identification of cataract among children is another challenge. Efforts are being undertaken to screen families where hereditary cataract has been detected.

Retaining trained staff continues to be a big challenge. The current strategy is to undertake continuous training of VTs to fill the gaps, and advocacy to change institutional policies in favour of retention. Refresher courses are taking place to improve quality of services.

Making Vision Centres sustainable is currently the toughest challenge. The performance of each centre has been systematically analyzed and attention has been given to strengthening the weaker ones. Emphasis is being placed on increasing uptake of services through better services, increasing the number of spectacles sold, and IT based monitoring of activities. Continuation of service activities beyond the project period mostly depends on the sustainability of these units.

Planning an eye care project in a relatively inaccessible geographic region needs special consideration. An effort should be made to select and train workers from the same region. While budgeting, a significant amount should be allotted for transport. This kind of project can never be a remotely managed one. Active participation of first and second tiers of leadership is very essential for monitoring, motivating field staff, deepening the relationship with the community and overall sustainability.

**Figure:**
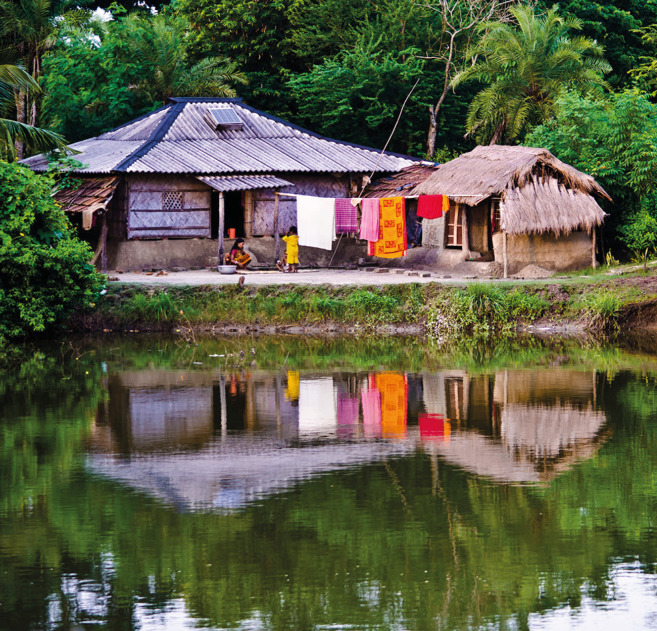
Sunderbans is an area of extreme poverty and ill health exacerbated by access difficulties © Joydip Roy CC BY

**Table:** Progress against planned output for 01 October 2015 to 31 March 2016:

Output type	Target	Planned Outputs			Actual outputs to date			Variance%
		**M**	**F**	**Total**	**M**	**F**	**Total**	
**PATIENTS**								
**Surgeries (per eye)**								
Cataract surgery: adults	27,000	5,233	5,567	10,800	5,751	6,045	11,796	109
Good outcome VA >6/18	80%	4,606	4,868	9474	4,415	4,155	8,570	90
Cataract surgery: children	200	39	41	80	29	13	42	53
**Screening**								
School screening (1,308 schools)	457,800 children	114,456	128,871	243,327	98,676	111,543	210,219	86
Adult RE screening	330,000	56,663	59,887	116,550	59,770	61,076	120,846	104
**Refraction**								
Refractions/prescriptions (adults):	87,000	18,037	17,563	35,600	31,487	30,781	62,268	175
Spectacles prescribed (adults):	43,200	8,600	9,128	17,728	15,599	16,160	31,759	179
Free spectacles supplied (adults):	3,844	689	785	1,474	757	848	1,605	109
Spectacles supplied (children):	9,156	1,541	1,787	3,328	1,224	1,325	2,549	77
